# 3M microfoam™ surgical tape prevents nasal pressure injury associated with nasotracheal intubation: A randomized double-blind trial

**DOI:** 10.1097/MD.0000000000032679

**Published:** 2023-01-13

**Authors:** Mayumi Hashimoto, Aiji Sato (Boku), Yoshiki Sento, Yuji Kamimura, Eisuke Kako, Masahiro Okuda, Naoko Tachi, Yoko Okumura, Izumi Kuroda, Hiroshi Hoshijima, Hidekazu Ito, Kazuya Sobue

**Affiliations:** a Department of Anesthesiology, Aichi Gakuin University School of Dentistry 2-11 Suemori-dori, Chikusa-ku, Nagoya, Aichi, Japan; b Department of Anesthesiology and Intensive Care Medicine, Nagoya City University Graduate School of Medical Sciences 1 Kawasumi, Mizuho-cho, Mizuho-ku, Nagoya, Aichi, Japan; c Division of Dento-oral Anesthesiology, Tohoku University Graduate School of Dentistry 4-1 Seiryomachi, Aoba, Sendai, Miyagi, Japan; d Department of Anesthesiology, Toyokawa City Hospital, 23 Yahatacho Noji, Toyokawa-city, Aichi, Japan.

**Keywords:** nasal pressure injury, nasotracheal intubation, surgical tape

## Abstract

3M microfoam™ surgical tape (3ST: 3M Japan Limited) is used for pressure wound control of medical equipment. It is cushioned and can be fitted to any body part. Here we investigated whether 3ST prevents nasal pressure injury associated with nasotracheal intubation (NTI).

**Methods:**

We conducted a prospective, randomized double-blind study, enrolling 63 patients aged 20 to 70 years, who underwent general anesthesia with NTI. They were divided into 2 groups; those treated with 3ST (group *S*; n = 31) and control (group *C*; n = 31). After NTI and before securing the nasotracheal tube, a 35 × 25 mm 3ST was used to protect the nasal wing in group *S*, and the nasotracheal tube was fixed in place after NTI without protection in group *C*. The primary outcome was the presence or absence of nasal pressure injury after extubation. The Chi-Square test was used to assess the association between the 2 categorical variables.

**Results:**

Nasal pressure injury was observed in 7 and 19 patients from groups *S* and *C*, respectively, representing a significant difference between the 2 groups (24.1% vs 67.8%, *P* = .001). Remarkably, none of the patients developed ulcers.

**Conclusion:**

3ST prevents nasal pressure injury associated with NTI.

## 1. Introduction

Nasotracheal intubation (NTI) is frequently necessary during dental and oral maxillofacial surgeries, specifically during operations with operative field and airway converge. However, some complications associated with NTI such as nasal bleeding,^[1,2]^ bacteremia,^[3]^ retropharyngeal perforation,^[4]^ and pressure ulcers or necrosis of nasal alar have been reported.^[5–8]^ Furthermore, nasal pressure injury, including pressure ulcers or necrosis, during NTI occurs in 10% to 50% of cases^[5–8]^ and can lead to many postoperative issues such as persistent pain, cosmetic issues, and persistent treatment.

The National Pressure Ulcer Advisory Panel, European Pressure Ulcer Advisory Panel, and Pan-Pacific Pressure Injury Alliance collectively published *The Prevention and Treatment of Pressure Ulcers*.^[9]^ They revised the definition and changed the term “pressure ulcers” to “pressure injuries” in 2016 and included medical device-related pressure injuries and mucosal membrane pressure injuries in the category.^[10]^ According to several reports, nasotracheal tubes are closely associated with medical device-related pressure injuries.^[11–13]^

It is reported that the application of hydrocolloid dressing to the nasal alar is effective in preventing nasal pressure injury associated with NTI.^[5,6,8,14]^ However, hydrocolloid dressing is relatively expensive and difficult to use owing to its product form.

Hoshijima et al state that the meta-analysis of nasal protection strategy suggests that the use of a nasal protection strategy considerably reduces the risk of a nasal pressure injury during NTI; however, the number of samples in the meta-analysis was too small for trial sequential analysis, thus further research is needed.^[15]^ Therefore, there is still a need to establish new strategies for preventing nasal pressure injury associated with NTI. Surgical tape can be considered an alternative to hydrocolloid dressing. 3M microfoam™ surgical tape (3ST: 3M Japan Limited) is used for pressure wound control of medical equipment; moreover, it is cushioned and can be fitted to any part of the body. Additionally, it has the advantage of being relatively inexpensive compared to hydrocolloid products, and to our knowledge, there are no prospective randomized trials in the literature on the use of 3ST for preventing nasal pressure injury associated with NTI. Therefore, we hypothesized that 3ST prevents nasal pressure injury associated with NTI and decided to investigate this hypothesis.

## 2. Materials and methods

### 2.1. Ethics approval and consent to participate

This study was conducted in accordance with the ethical standards of the Declaration of Helsinki (1964) and its subsequent amendments. Moreover, this study adhered to the Consolidated Standards of Reporting Trials guidelines (CONSORT) and was approved by the Ethics Committee at the School of Dentistry, Aichi Gakuin University (Approval No. 637). Furthermore, written informed consent was obtained from all patients participating in the trial, and before patient enrollment, the trial was registered as a clinical trial at UMIN-CTR (Registration No. UMIN000045524, Date of first registration: 21/09/2021). The first patient was recruited and registered on September 27^th^, 2021.

### 2.2. Study design and population

**Figure 1.**
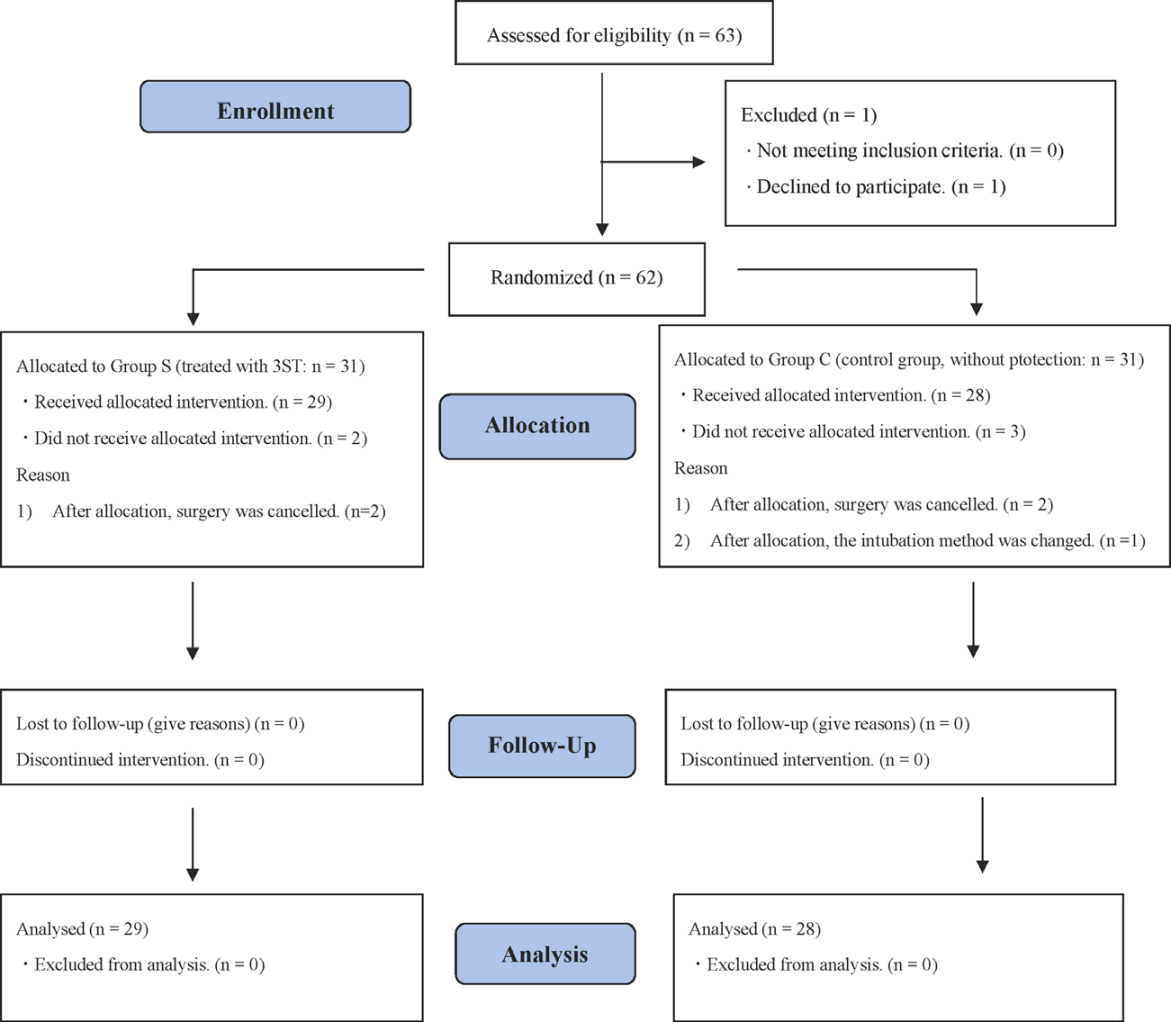
CONSORT flowchart describing patient recruitment.

We conducted a prospective, randomized double-blind study with a blinded evaluator and patients, enrolling 63 patients aged 20 to 70 years and scheduled to undergo general anesthesia with NTI for oral and maxillofacial surgery. Exclusion criteria were those with

an obvious nostril constriction (n = 0) and deformities inside the nostrils (n = 0) observed during preoperative CT test taken prior to the surgery, previous surgery around the nostrils (n = 0), and those prone to skin irritation (n = 0). Among the recruited patients, those who did not provide consent (n = 1) were excluded from the study. Therefore, the final study population included 62 patients who were randomly divided into 2 groups, namely the group treated with 3ST (group *S*: n = 31) and the control group (group *C*: n = 31). Patients were assigned randomly to each group using computer-generated random number (Fig. 1).

### 2.3. Anesthesia and intubation methods

**Figure 2.**
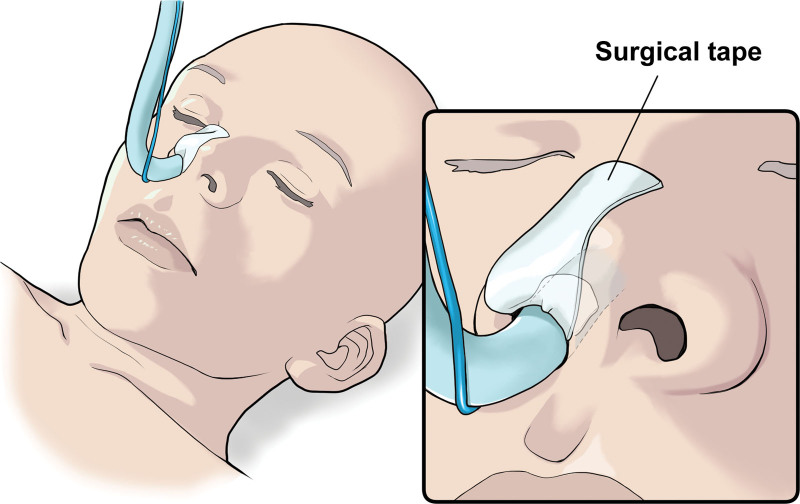
Protection method of nasal wing. Group *S* used a 35-mm wide and 35-mm long 3ST to protect the nasal wing. The vertical 1/3 of 3ST was bent toward the nasal cavity using dental tweezers. 3ST = 3m microfoamTM surgical tape.

The same method of anesthesia was employed for all patients. The standard vital signs monitors (electrocardiogram, blood pressure, and oxygen saturation) were inspected. Anesthesia was induced using propofol (3 *μ*g/mL target control infusion), remifentanil (0.2 *µ*g/kg/minutes), and fentanyl (100 *µ*g) with rocuronium (0.6 mg/kg) used as a neuromuscular blocking agent. Subsequently, mask ventilation was applied at 100% oxygen with propofol and remifentanil, and the patients’ nasal mucosa and inferior nasal passages were adequately disinfected using benzalkonium (ZALKONIN^®^ SOLUTION 0.025, Kenei Pharmaceutical Co., Ltd, Osaka).^[16]^ Tramazoline was used for hemostasis during NTI.^[17]^ Following this, NTI through the right nostril was performed to reduce the risk of nasal bleeding.^[18]^ Subsequently after NTI and before securing the nasotracheal tube, group *S* used a 35-mm wide and 35-mm long 3ST to protect the nasal wing. The vertical 1/3 of 3ST was bent toward the nasal cavity using dental tweezers (Fig. 2). In group *C*, the nasotracheal tube was fixed in place after NTI without protection. The nasotracheal tube used in this study was Polar™ Preformed Tracheal Tube (Smith Medical Japan Ltd, Tokyo), and the tube size was ID 7.0 mm for men and ID 6.5 mm for women.

### 2.4. Measurements

The primary outcome was the presence or absence of nasal pressure injury observed from the nasal tip to the nasal alar. After extubation, an assessment was performed by the operating room nurse, who was not informed whether the patient belonged to group *S* or *C*. All patients were evaluated. The secondary outcome was the presence or absence of an ulcer. Patient background, operative time, anesthesia time, and operative details were also evaluated.

Furthermore, we compared the price of 3ST used in this study and the hydrocolloid dressing that can currently be used for pressure ulcer prevention in Japan.^[19]^

### 2.5. Statistical analysis

We estimated that a minimum sample of 56 patients would be needed, where the threshold response, *α*error, and power (1−*β*) were set at 40%, 0.05%, and 0.80% respectively. Threshold response was calculated based on the statistical results of a pilot study where the patient distribution in the amount of nasal pressure injury of the nasal tip to the nasal alar after extubation was used as a standard (group *S*, n = 10; group *C*, n = 10).

Since the use of statistical tests in the absence of reliable sample size calculation decreases its weightage, we calculated our final sample size considering an expected dropout rate of 0.05 based on our pilot study. Hence, if a dropout rate (R) is expected, a simple but adequate adjustment is provided by N_d_ = N/ (1 − R)^2^, where N is the sample size calculated assuming no dropout and N_d_ is the estimated sample size required when dropouts are expected.^[20]^ Therefore, after adjusting for dropouts, a final sample of 62 patients was recruited.

For statistical testing, the Chi-square test of independence was used to test the relationship between 2 categorical variables, and the Mann–Whitney *U* test was used to compare differences between 2 independent groups for continuous variables. The 2-sided statistical significance level was set at *P* ≤ .05. Statistical analysis of recorded data was performed using IBM^®^ SPSS^®^ Statics Ver26.

## 3. Results

### 3.1. Patients’ background

Sixty-three patients were selected to participate in this study from September 2021 to November 2021. The participant CONSORT flow diagram is presented in Figure 1. As 1 patient declined to participate in the study, a total of 62 patients were randomly assigned into 2 groups based on the prevention method of nasal pressure injury. Moreover, 5 patients dropped out during the trial; thus, the final sample size was 57. Table 1 depicts the demographic characteristics, operation, and anesthesia time, and no statistical differences between the groups were observed.

**Table 1:** Patient demographic information.

	Group *S*	Group *C*	*P*-value
Male/Female	6/23	8/20	.49
Age (yr)	33 (25–52)	28.5 (24.75–32.5)	.52
Height (cm)	160 (156–169)	159 (155.5–165.25)	.89
Weight (kg)	53 (48–65)	55 (47.75–66.75)	.69
Operation time (min)	86 (54–141)	82 (56.5–127.25)	.69
Anesthesia time (min)	140 (87–187)	122 (90.75–162.25)	.69

Values are in numbers or median (quartiles 1–3).

### 3.2. Presence or absence of nasal pressure injury

As depicted in Table 2, the presence of nasal pressure injury from the nasal tip to the nasal alar was observed in 7 and 19 patients from groups *S* and *C*, respectively, representing a significant difference in the proportion in the 2 groups (24.1% [7/29] vs 67.8% [19/28], *P* = .001). None of the patients developed ulcers.

**Table 2:** Distribution of the patients based on nasal pressure injury associated with nasotracheal intubation.

	Group *S*	Group *C*	*P*-value
None	22	9	.001
Nasal pressure injury	7	19	
Ulcer	0/7	0/19	

Values are in numbers.

### 3.3. Comparison of the price of the surgical tape and hydrocolloid formulation

Table 3 shows a comparison of the price of 3ST used in this study and the hydrocolloid dressing. In this study, the cost per patient for 3ST was approximately 3.6 yen. Conversely, the cost per patient for hydrocolloid dressing ranges from 350 yen to 1000 yen, although there are differences depending on the product.

**Table 3:** Comparison of the surgical tape prices and hydrocolloid formulation (1 USD = 115 yen).

Type of product	Product name	Minimum size	Quantity	List price	Price per patient for use in this study or if used
Surgical tape	3M microform™ surgical tape	25 mm (length) × 5000 mm (width)	12 rolls	6000 yen (500 yen/roll)	3.6 yen
					(5000 mm ÷ 35 mm = 140
					500 yen ÷ 140 = 3.6 yen)
Hydrocolloid	ABSOCURE® -SURGICAL	50 mm (length) × 100 mm (width)	20 sheets	7000 yen (350 yen/sheet)	350 yen (if used)
	tegaderm™ hydrocolloid Thin	100 mm × 120 mm (oval)	10 sheets	3640 yen (364 yen/sheet)	364 yen (if used)
	Duoactive ® ET	50 mm (length) × 100 mm (width)	20 sheets	6000 yen (300 yen/sheet)	300 yen (if used)
	ABSOCURE ®–WOUND	100 mm (length) × 100 mm (width)	5 sheets	5000 yen (1000 yen/sheet)	1000 yen (if used)
	Tegaderm™ Hydrocolloid	100 mm × 120 mm (oval)	5 sheets	3120 yen (624 yen/sheet)	624 yen (if used)

## 4. Discussion and conclusion

In this study, on using 3ST for preventing nasal pressure injury associated with NTI, we observed a significant difference in the frequency of nasal pressure injury from the nasal tip to the nasal alar between the groups. Generally, nasal pressure injury during NTI is caused by local ischemia between the nasal columella and alar because of the continuous pressure exerted by the tracheal tube. Moreover, nasotracheal tubes are closely associated with medical device-related pressure injuries.^[11–13]^ Medical device-related pressure injuries are a remarkable public health concern, particularly as these injuries affect patients’ wellbeing and increase the cost of care for both patients and healthcare providers.^[13]^ Furthermore, the incidence of medical device-related pressure injuries in hospitalized patients should be considered a negative point in terms of the performance of healthcare providers.^[11]^ Hence, based on the points discussed above, it is crucial to prevent medical device-related pressure injuries.

In previous studies on the use of hydrocolloid dressing for the prevention of nasal pressure injury associated with NTI, the preventive effect ranged from 56.7% to 95%.^[5,6,8]^ The preventive effect of the present study using 3ST was 75.9% (22/29). Although it is impossible to make a simple comparison, 3ST was found to have a preventive effect as good as that of hydrocolloid dressing.

3ST is a multidirectional, stretchable, and thick dressing. It is conformable and adheres gently yet securely to uneven surfaces. Moreover, it is water resistant, free of natural rubber latex, and hypoallergenic foam-based elastic tape. However, as 3ST has strong adhesiveness, the possibility of the adhesive substance getting on the skin cannot be denied. Nevertheless, it is considered a suitable tape for pressure and dressing fixation.

As shown in Table 3, hydrocolloid dressing products are relatively more expensive than 3ST. It is important to keep the cost low, as this tape will be used for prevention and considering that it is important for the medical economy. In this study, a 35-mm wide and 25-mm long 3ST was used. 3ST is a tape measure, which is economical and cautious. Most of the hydrocolloid dressings are in the form of centimeters by centimeters, which makes them extremely wasteful. Hence, it is better to use 3ST for NTI-related nasal pressure injuries, and the results of this study indicate that surgical tape can be used in place of hydrocolloid products.

Other risk factors for nasal pressure injury during NTI included gender, prolonged operating time, and long intensive care unit stay.^[6]^ Concerning gender, in the present study, the NTI caused nasal pressure injury to 2 out of 6 males in group *S* and 4 out of 8 males in group *C*. Further, the NTI caused nasal pressure injury in 5 out of 23 females in group *S* and 15 out of 20 females in group *C*. As for the operation time, some cases of nasal pressure injury occurred despite the short operation time in both groups. Although these results were inconsistent with previous studies, this may be attributed to the fact that the sample size was too small to assess gender, time, and other factors.

This study has several limitations. First, several anesthesiologists were involved in the tape fixation in both groups. Although the tape fixation methods were standardized to the maximum possible extent before initiating the study, individual differences were undeniable. Second, several healthy young patients participated in this study, and we do not know whether the results of this study apply to all age groups. Finally, this study is not a direct comparison between 3ST and hydrocolloid dressing. Future comparative studies between 3ST and hydrocolloid dressing are warranted. In conclusion, 3ST prevents nasal pressure injury associated with NTI. In addition, using 3ST is less expensive than hydrocolloid dressing.

## Acknowledgements

The authors would like to thank Enago (www.enago.jp) for the English language review.

## Author contributions

**Conceptualization:** Mayumi Hashimoto, Aiji Sato (Boku), Yoshiki Sento, Yuji Kamimura, Eisuke Kako, Masahiro Okuda, Naoko Tachi, Yoko Okumura, Izumi Kuroda, Hiroshi Hoshijima, Hidekazu Ito, Kazuya Sobue.

**Data curation:** Mayumi Hashimoto, Aiji Sato (Boku), Masahiro Okuda, Naoko Tachi, Yoko Okumura, Izumi Kuroda.

**Formal analysis:** Aiji Sato (Boku), Yoshiki Sento, Yuji Kamimura, Eisuke Kako, Naoko Tachi, Hiroshi Hoshijima, Hidekazu Ito.

**Investigation:** Mayumi Hashimoto, Aiji Sato (Boku).

**Supervision:** Kazuya Sobue.

**Writing – original draft:** Mayumi Hashimoto, Aiji Sato (Boku), Hiroshi Hoshijima, Hidekazu Ito.

**Writing – review & editing:** Kazuya Sobue.
